# Measurement of brainstem diameter in small-breed dogs using magnetic resonance imaging

**DOI:** 10.3389/fvets.2023.1183412

**Published:** 2023-07-13

**Authors:** Jihyun Kim, Danbee Kwon, Sung-Soo Kim, Kichang Lee, Hakyoung Yoon

**Affiliations:** ^1^Department of Veterinary Medical Imaging, College of Veterinary Medicine, Jeonbuk National University, Iksan, Republic of Korea; ^2^Bundang Leaders Animal Medical Center, Seongnam-si, Republic of Korea; ^3^VIP Animal Medical Center, Seoul, Republic of Korea

**Keywords:** canine, brainstem, midbrain, pons, medulla oblongata, size, age, body weight

## Abstract

Measurement of brainstem diameters (midbrain, pons, and medulla oblongata)is of potential clinical significance, as changes in brainstem size may decrease or increase due to age, neurodegenerative disorders, or neoplasms. In human medicine, numerous studies have reported the normal reference range of brainstem size, which is hitherto unexplored in veterinary medicine, particularly for small-breed dogs. Therefore, this study aims to investigate the reference range of brainstem diameters in small-breed dogs and to correlate the measurements with age, body weight (BW), and body condition score (BCS). Herein, magnetic resonance (MR) images of 544 small-breed dogs were evaluated. Based on the exclusion criteria, 193 dogs were included in the midbrain and pons evaluation, and of these, 119 dogs were included in the medulla oblongata evaluation. Using MR images, the height and width of the midbrain, pons, and medulla oblongata were measured on the median and transverse plane on the T1-weighted image. For the medulla oblongata, two points were measured for each height and width. The mean values of midbrain height (MH), midbrain width (MW), pons height (PH), pons width (PW), medulla oblongata height at the fourth ventricle level (MOHV), medulla oblongata height at the cervicomedullary (CM) junction level (MOHC), rostral medulla oblongata width (RMOW), and caudal medulla oblongata width (CMOW) were 7.18 ± 0.56 mm, 17.42 ± 1.21 mm, 9.73 ± 0.64 mm, 17.23 ± 1.21 mm, 6.06 ± 0.53 mm, 5.77 ± 0.40 mm, 18.93 ± 1.25 mm, and 10.12 ± 1.08 mm, respectively. No significant differences were found between male and female dogs for all the measurements. A negative correlation was found between age and midbrain diameter, including MH (p < 0.001) and MW (*p*  = 0.002). All brainstem diameters were correlated positively with BW (*p*  < 0.05). No significant correlation was found between BCS and all brainstem diameters. Brainstem diameters differed significantly between breeds (*p*  < 0.05), except for MW (*p*  = 0.137). This study assessed linear measurements of the brainstem diameter in small-breed dogs. We suggest that these results could be useful in assessing abnormal conditions of the brainstem in small-breed dogs.

## 1. Introduction

The brainstem—the caudoventral part of the cranial cavity, located between the diencephalon, cerebellum, and spinal cord (1, 2)—is composed of the midbrain (mesencephalon), pons (ventral metencephalon), and medulla oblongata (myelencephalon), and most of the cranial nerves originate from nuclei located in the midbrain through the medulla oblongata (1–6). The brainstem is the caudoventral part of the cranial cavity, located between the diencephalon, cerebellum, and spinal cord (1, 2). The brainstem plays an important role in the regulation of posture, consciousness, respiratory, and cardiovascular coordination (3–6). Therefore, clinical signs associated with brainstem dysfunction include disturbances of consciousness, gait abnormalities, cranial nerve deficits, postural abnormalities, abnormal respiratory activities, and autonomic dysfunction (3).

Magnetic resonance imaging (MRI) is a non-invasive modality that is widely used to detect brain lesions, including in the brainstem (7, 8). MRI provides accurate and detailed quantitative morphological information about the brainstem (5, 6, 9, 10). In previous human studies, linear measurement has been suggested as a reliable and rapid method for evaluating brainstem size, and a multitude of studies measuring the brainstem quantitatively have been reported (6, 9–16).

Studies establishing normal reference ranges of brainstem diameters in humans through linear measurements using MRI have been carried out (5, 9–15). Brainstem size has been found to decrease with the normal aging process (5, 9, 11–14). Furthermore, brainstem diameters decrease with neurodegenerative diseases, such as Parkinson’s disease, progressive supranuclear palsy, or other atrophic processes (6, 16). Contrastingly, some diffusely infiltrating brain tumors increase the diameter of the brainstem with no marked signal change in the MR images (16). Consequently, knowledge of the morphology of the normal brainstem is crucial, not only to understand normal age-related degeneration but also to compare the pathophysiology of neurodegenerative disorders and detect neoplastic changes (11, 16).

Quantitative measurement of brainstem diameters in dogs using MRI has not yet been studied in veterinary medicine, and thus, there is a lack of normative data pertaining to the brainstem, particularly for small-breed dogs. Consequently, the aim of this study is to: (i) establish a reference range for brainstem diameters (midbrain, pons, and medulla oblongata) in small-breed dogs; (ii) analyze the statistical differences in brainstem diameters between sex and different breeds; and (iii) analyze the correlation between brainstem diameters and body weight (BW), body condition score (BCS), and age.

## 2. Materials and methods

### 2.1. Animals

This was a retrospective multicenter study. The medical records and brain MR images of 544 small-breed dogs who visited the Leaders Animal Medical Center and VIP Animal Medical Center from June 2019 to December 2022 were collected. Additionally, detailed medical information about each dog, including breed, sex, age, and BW, was collected. The following exclusion criteria were applied: presence of lesions in the parenchyma of the brainstem, any lesion in the brain parenchyma associated with neoplasia, inflammation, edema, hemorrhage, or infarction, clinically significant Chiari-like malformation or abnormalities at the craniocervical junction with or without syringomyelia, severe ventriculomegaly, or hydrocephalus. Consequently, 193 dogs were included for midbrain and pons measurement. Of these, 74 dogs with mild abnormalities at the craniocervical junctions were excluded, and 119 dogs were included for medulla oblongata measurement.

For the 193 dogs included in the midbrain pons measurement, the sex distribution was castrated males (*n* = 99), intact males (*n* = 6), spayed females (*n* = 74), and intact females (*n* = 14); the breed distribution was Chihuahua (*n* = 12), Maltese (*n* = 59), Mixed (*n* = 18), Pomeranian (*n* = 35), Poodle (*n* = 33), Shih Tzu (*n* = 20), and Yorkshire Terrier (*n* = 16); the mean age was 8.30 ± 3.95 (0.58–17.00) years; and the mean BW was 4.11 ± 1.67 kg (1.45–9.55 kg). For the 119 dogs included in the medulla oblongata measurement, the sex distribution was castrated males (*n* = 63), intact males (*n* = 5), spayed females (*n* = 41), and intact females (*n* = 10); the breed distribution was Chihuahua (*n* = 9), Maltese (*n* = 35), Mixed (*n* = 11), Pomeranian (*n* = 14), poodle (*n* = 22), Shih Tzu (*n* = 19), and Yorkshire Terrier (*n* = 9); the mean age was 8.47 ± 4.06 (0.67–17.00) years; and the mean BW was 4.53 ± 1.75 (1.45–9.55) kg. This study was approved by the Institutional Animal Care and Use Committee of Jeonbuk National University (Approval No. NON 2022-086).

### 2.2. Measurements

MR images of the brain were acquired using 1.5 Tesla MRI machines (GoldSeal Signa HDxt 1.5T, GE Healthcare, United States, and Signa Creator 1.5T, GE Healthcare, United States). In the study, the median and transverse planes of T1-weighted images (slice thickness: 2.5 or 3 mm, repetition time: TR = 400–1,790 ms, TE = 9–25 ms) were used for measurement. The median plane of the T1-weighted image was used for height measurement and the transverse plane of the T1-weighted image was used for width measurement.

**Figure 1:**
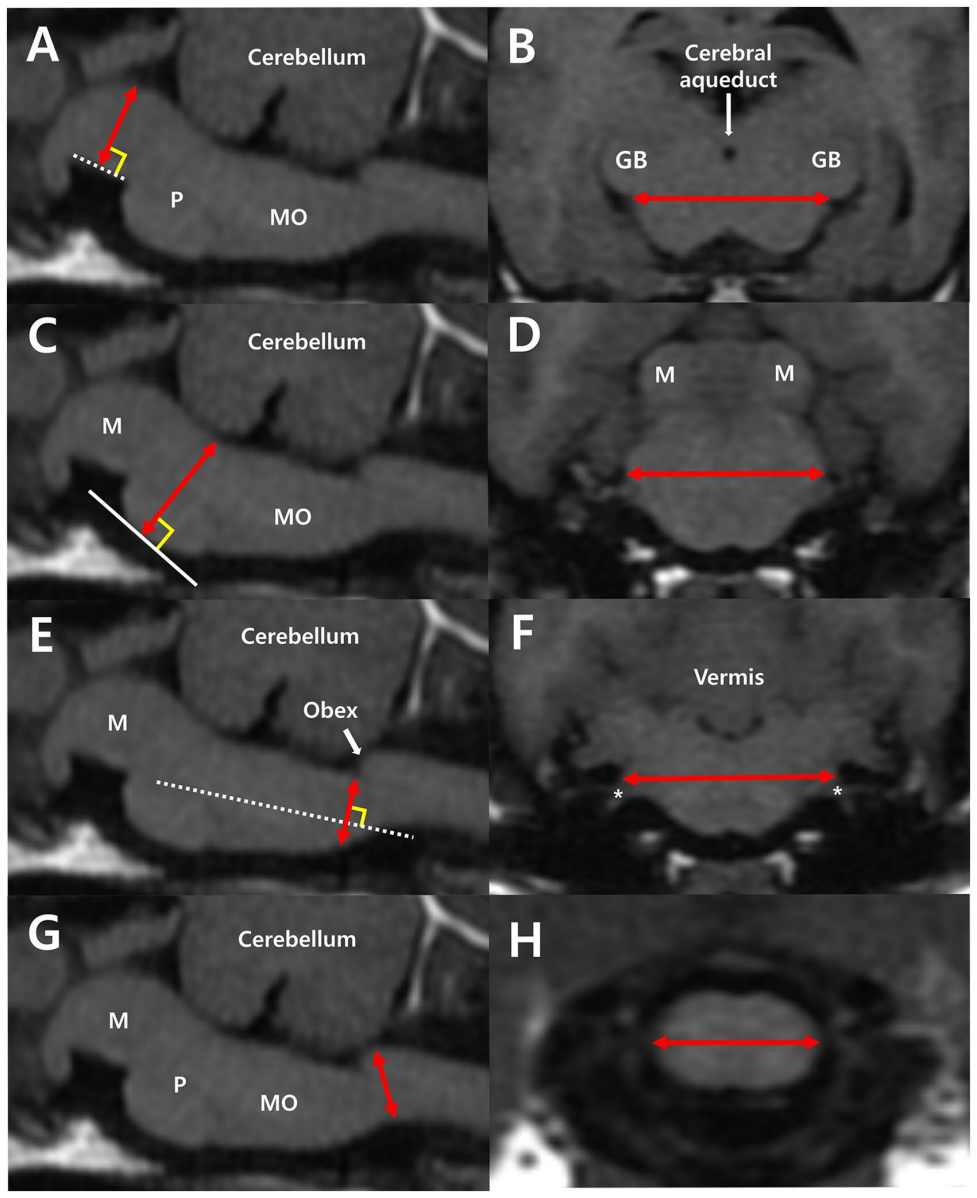
Median plane of the brainstem **(A,C,E,G)** and transverse plane of the midbrain **(B)**, pons **(D)**, and medulla oblongata **(F,H)** in T1-weighted MRI images. **(A)** Midbrain height (MH) was measured from the midpoint of the interpeduncular fossa to the margin of the cerebral aqueduct, perpendicular to the interpeduncular fossa (dotted line). **(B)** Midbrain width (MW) was measured by connecting both bilateral commissures of the medial geniculate body and the brachium of the caudal colliculus. **(C)** Pons height (PH) was measured from the ventral margin of the pons to the margin of the fourth ventricle, perpendicular to the tangent line (white line) of the most ventral point of the pons. **(D)** Pons width (PW) is the maximum width connecting both bilateral middle cerebellar peduncles. **(E)** Medulla oblongata height at the fourth ventricle level (MOHV) was measured from the kinking point at the level of the obex to the ventral margin of the medulla, perpendicular to the long axis of the medulla. **(F)** Rostral medulla oblongata width (RMOW) was measured from one end to the other of the bilateral cochlear nuclei. CN VIII (asterisk), the vestibulocochlear nerve, extends from the cochlear nuclei. **(G)** Medulla oblongata height at the CM junction (MOHC) is the diameter measured at the level of the CM junction. **(H)** Caudal medulla oblongata width (CMOW) is the widest width of the medulla at the level of the CM junction. M, midbrain; P, pons; MO, medulla oblongata; GB, geniculate body.

Linear measurements of height and width of each of the following were performed: midbrain, pons, and medulla oblongata (Figure 1). For the midbrain, midbrain height (MH) was measured from the midpoint of the interpeduncular fossa to the margin of the cerebral aqueduct, perpendicular to the line of the interpeduncular fossa (Figure 1A). Midbrain width (MW) was measured by connecting both bilateral commissures of the medial geniculate body and brachium of the caudal colliculus (Figure 1B). For the pons, pons height (PH) was measured from the ventral margin of the pons to the margin of the fourth ventricle, perpendicular to the tangent line of the most ventral point of the pons (Figure 1C). Pons width (PW) was calculated by measuring the maximum width connecting both bilateral middle cerebellar peduncles (Figure 1D). The diameter of the medulla oblongata was measured at two different levels, one for height and one for width. The height of the medulla oblongata was measured at the fourth ventricle level and cervicomedullary (CM) junction level. The medulla oblongata height at the fourth ventricle level (MOHV) was measured from the kinking point at the level of the obex to the ventral margin of the medulla, perpendicular to the long axis of the medulla (Figure 1E). Medulla oblongata height at the CM junction (MOHC) was measured at the level of the CM junction (Figure 1G). The width of the medulla oblongata was measured at the rostral region and caudal region. Rostral medulla oblongata width (RMOW) was measured from end to end of the bilateral cochlear nuclei at the trapezoid level (Figure 1F). Caudal medulla oblongata width (CMOW) was obtained by measuring the greatest width of the medulla at the level of the CM junction (Figure 1H).

For intra-observer reliability analysis, measurements of all brainstem diameters (193 dogs for the midbrain and pons and 119 dogs for the medulla oblongata) were repeated twice by observer A (JK), and the mean values were used for all statistical analysis. For inter-observer reliability analysis, all brainstem diameters (193 dogs for the midbrain and pons; and 119 dogs for the medulla oblongata) were measured by observers A and B (Residents of the Veterinary Medical Imaging Department of the Teaching Hospital of Jeonbuk National University).

### 2.3. Statistics

All brainstem diameters are presented as mean ± standard deviation (SD). Linear regression analysis was used to analyze the correlation between brainstem diameter and “age, BW, and BCS.” An Independent *t*-test was used to analyze the mean differences in brainstem diameter between sexes. One-way ANOVA was used to analyze the differences in brainstem diameter between breeds. Intra-observer and inter-observer reliability for all measurements was assessed using an absolute agreement-type intraclass correlation coefficient (ICC) with 95% confidence intervals (CI). IBM SPSS Statistics (version 27.0; IBB Corp., Armonk, NY, United States) was used for all the statistical analyses, and all experimental values were considered to be statistically significant at *p <* 0.05.

## 3. Results

Mean ± standard deviations of MH, MW, PH, PW, MOHV, MOHC, RMOW, and CMOW are summarized in Table 1.

**Table 1:** Mean and standard deviations of brainstem diameters.

Variables	*N*	Age (years)	BW (kg)	Mean ± SD (mm) (range)
MH	193	8.30 ± 3.95 (0.58–17.00)	4.11 ± 1.67 (1.45–9.55)	7.18 ± 0.56 (4.80–8.70)
MW				17.42 ± 1.21 (12.30–20.10)
PH				9.73 ± 0.64 (7.76–11.60)
PW				17.23 ± 1.21 (13.65–20.60)
MOHV	119	8.47 ± 4.06 (0.67–17.00)	4.53 ± 1.75 (1.45–9.55)	6.06 ± 0.53 (4.80–7.44)
MOHC				5.77 ± 0.40 (4.95–6.98)
RMOW				18.93 ± 1.25 (15.9–23.95)
CMOW				10.12 ± 1.08 (8.17–14.05)

BW, body weight; SD, standard deviation; MH, midbrain height; MW, midbrain width; PH, pons height; PW, pons width; MOHV, medulla oblongata height at the fourth ventricle level; MOHC, medulla oblongata height at the CM junction; RMOW, rostral medulla oblongata width; CMOW, caudal medulla oblongata width.

### 3.1. Differences in brainstem diameter between sexes

The dogs were divided into two groups of males and females [for the midbrain and pons, males (*n* = 105), females (*n* = 88); for the medulla oblongata, males (*n* = 68), females (*n* = 51)]. Mean ± SD values of all brainstem diameters for each sex and the analysis of difference of each value between sexes are summarized in Table 2. There was no statistically significant difference between sex in MH (*p* = 0.076), MW (*p* = 0.320), PH (*p* = 0.065), PW (*p* = 0.056), MOHV (*p* = 0.603), MOHC (*p* = 0.549), RMOW (*p* = 0.054), and CMOW (*p* = 0.702).

**Table 2:** Mean and standard deviation of all brainstem diameter values by sex and analysis of the difference in each value between sexes using an independent *t*-test.

	*N*	Mean ± SD (mm)		*p*-value
		Male	Female	
MH	Male (*n* = 105)	7.11 ± 0.61	7.25 ± 0.48	0.076
MW		17.50 ± 1.26	17.33 ± 1.14	0.320
PH	Female (*n* = 88)	9.65 ± 0.62	9.82 ± 0.67	0.065
PW		17.38 ± 1.22	17.04 ± 1.19	0.056
MOHV	Male (*n* = 68)	6.04 ± 0.46	6.09 ± 0.62	0.603
MOHC		5.79 ± 0.42	5.75 ± 0.38	0.549
	Female (*n* = 51)			
RMOW		19.12 ± 1.15	18.68 ± 1.35	0.054
CMOW		10.15 ± 0.98	10.07 ± 1.21	0.702

SD, standard deviation; MH, midbrain height; MW, midbrain width; PH, pons height; PW, pons width; MOHV, medulla oblongata height at the fourth ventricle level; MOHC, medulla oblongata height at the cervicomedullary junction; RMOW, rostral medulla oblongata width; CMOW, caudal medulla oblongata width.

### 3.2. Correlation between brainstem diameter and “age, BW, and BCS”

**Figure 2:**
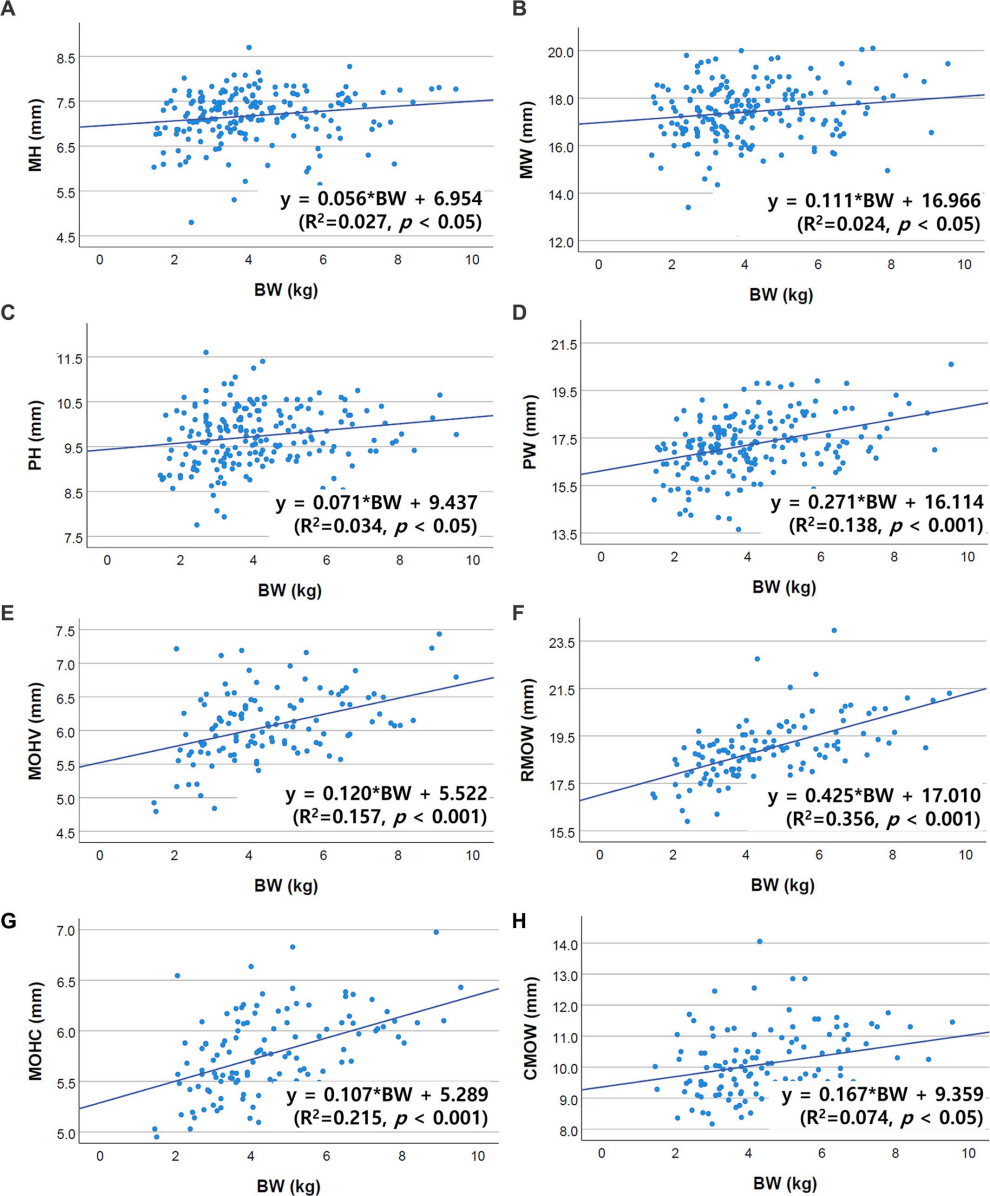
Scatter plots of the linear regression analysis of the relationship between BW and brainstem diameter. All brainstem diameters showed significant positive correlations with BW. **(A)** Positive correlation between MH and BW (*R*^2^ = 0.027, *p* < 0.05). **(B)** Positive correlation between MW and BW (*R*^2^ = 0.024, *p* < 0.05). **(C)** Positive correlation between PH and BW (*R*^2^ = 0.034, *p* < 0.05). **(D)** Positive correlation between PW and BW (*R*^2^ = 0.138, *p* < 0.001). **(E)** Positive correlation between MOHV and BW (*R*^2^ = 0.157, *p* < 0.001). **(F)** Positive correlation between RMOW and BW (*R*^2^ = 0.356, *p* < 0.001). **(G)** Positive correlation between MOHC and BW (*R*^2^ = 0.215, *p* < 0.001). **(H)** Positive correlation between CMOW and BW (*R*^2^ = 0.074, *p* < 0.05).

**Figure 3:**
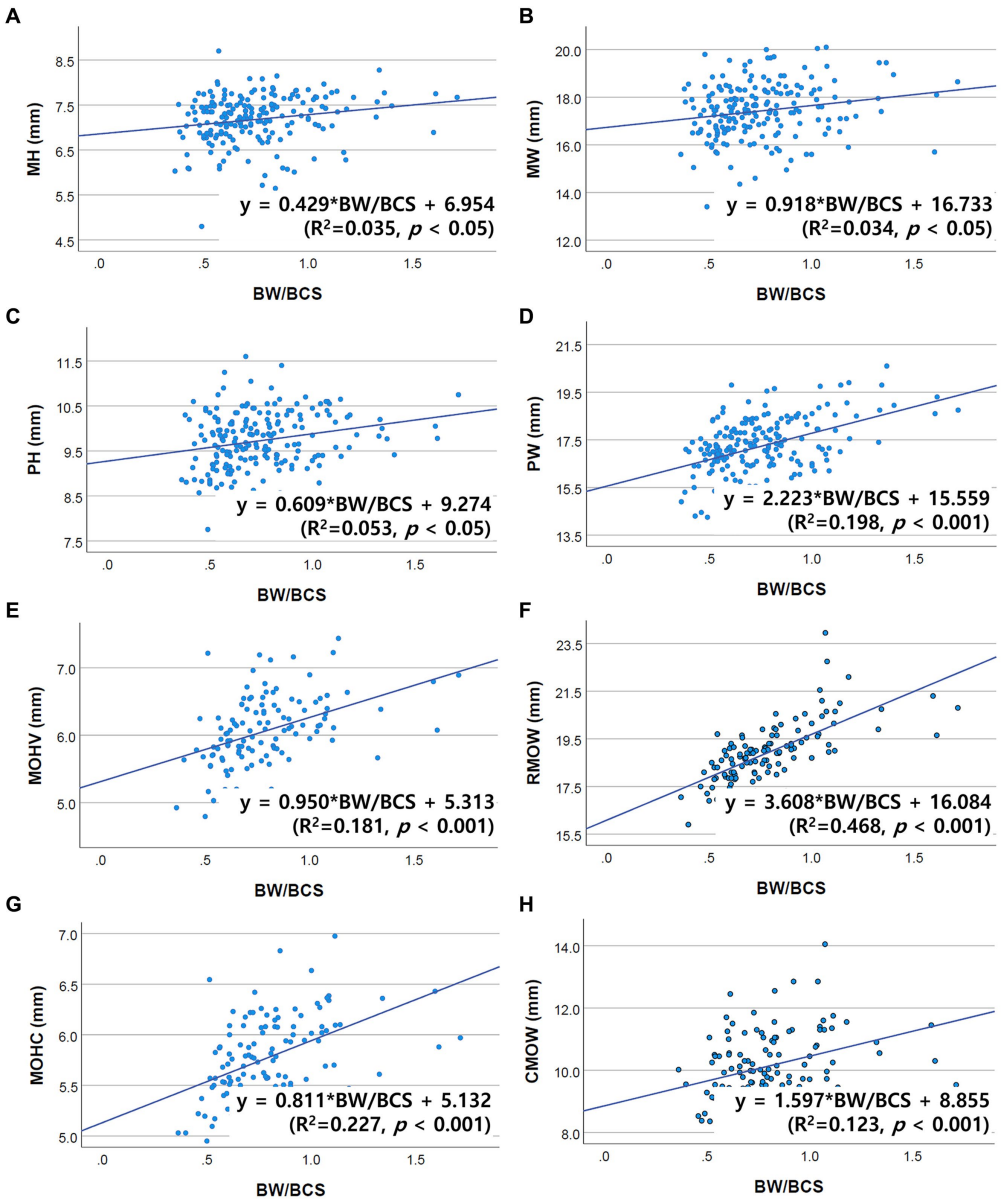
Scatter plots of the linear regression analysis of the relationship between the BW/BCS index and brainstem diameter. All brainstem diameters were significantly positively correlated with the BW/BCS index. **(A)** Positive correlation between MH and BW/BCS (*R*^2^ = 0.035, *p* < 0.05). **(B)** Positive correlation between MW and BW/BCS (*R*^2^ = 0.034, *p* < 0.05). **(C)** Positive correlation between PH and BW/BCS (*R*^2^ = 0.053, *p* < 0.05). **(D)** Positive correlation between PW and BW/BCS (*R*^2^ = 0.198, *p* < 0.001). **(E)** Positive correlation between MOHV and BW/BCS (*R*^2^ = 0.181, *p* < 0.001). **(F)** Positive correlation between RMOW and BW/BCS (*R*^2^ = 0.468, *p* < 0.001). **(G)** Positive correlation between MOHC and BW/BCS (*R*^2^ = 0.227, *p* < 0.001). **(H)** Positive correlation between CMOW and BW/BCS (*R*^2^ = 0.123, *p* < 0.001).

**Figure 4:**
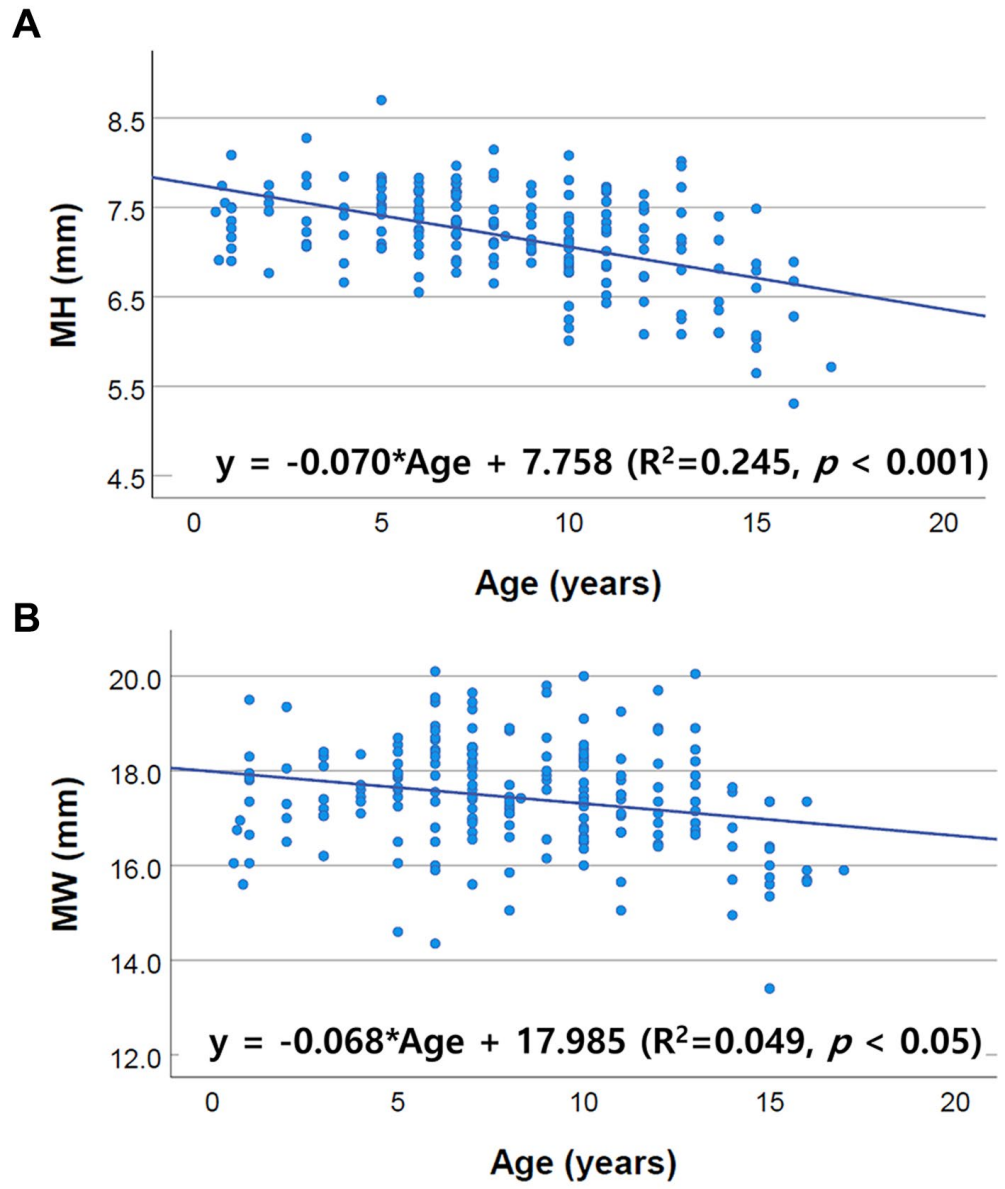
Scatter plots of the linear regression analysis of the relationship between age and brainstem diameter. Only MH and MW correlated negatively with age. The other values, including PH, PW, MOHV, MOHC, RMOW and CMOW, had no significant correlation with age. **(A)** Negative correlation between MH and age (*R*^2^ = 0.245, *p* < 0.001). **(B)** Negative correlation between MW and age (*R*^2^ = 0.049, *p* < 0.05).

Correlations between brainstem diameter and “age, BW, and BCS” were analyzed through simple linear regression analysis. Scatter plots and the results of linear regression analysis are presented in Figures 2–4. All the brainstem diameters positively correlated with BW: MH (*p* = 0.023), MW (*p* = 0.033), PH (*p* = 0.010), PW (*p* < 0.001), MOHV (*p* < 0.001), MOHC (*p* < 0.001), RMOW (*p* < 0.001), and CMOW (*p* = 0.003). On the other hand, none of the parameters correlated significantly with BCS. However, there was a significantly positive correlation between brainstem diameter and the BW/BCS index in all brainstem diameters, in contrast to the correlation between BCS alone. In the correlation between age and brainstem diameter, MH (*p* < 0.001) and MW (*p* = 0.002) were negatively correlated with age. PH, PW, MOHV, MOHC, RMOW, and CMOW were not significantly correlated with age.

### 3.3. Differences in brainstem diameters between breeds

**Figure 5:**
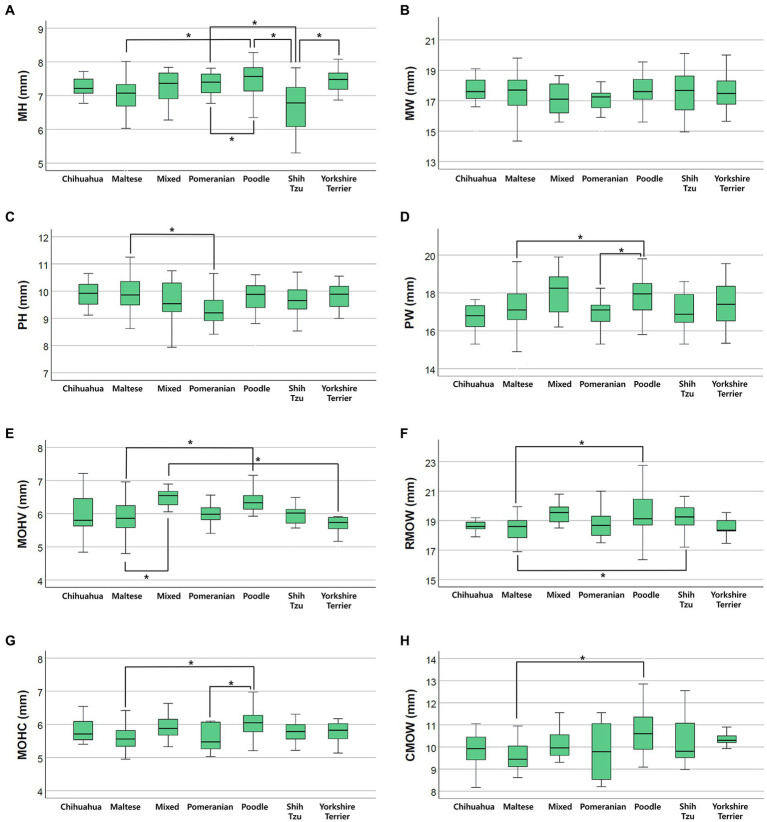
Box plots of the difference in brainstem diameters between breeds. There were statistically significant differences between breeds in **(A)** MH (*p* < 0.001), **(C)** PH (*p* = 0.006), **(D)** PW (*p* = 0.003), **(E)** MOHV (*p* < 0.001), **(F)** RMOW (*p* = 0.004), **(G)** MOHC (*p* < 0.001), and **(H)** CMOW (*p* = 0.008). There were no statistically significant differences between breeds in **(B)** MW (*p* = 0.137).

Differences in brainstem diameters were analyzed in seven small breeds (Chihuahua, Maltese, Mixed, Pomeranian, Poodle, Shih Tzu, and Yorkshire Terrier). Apart from MW (*p* = 0.137), the remaining values, including MH (*p* < 0.001), PH (*p* = 0.006), PW (*p* = 0.003), MOHV (*p* < 0.001), MOHC (*p* < 0.001), RMOW (*p* = 0.004), and CMOW (*p* = 0.008), had significant differences between breeds (Figure 5). Mean ± standard deviations of brainstem diameters categorized by breed are summarized in Tables 3 4.

**Table 3:** Mean and standard deviations of midbrain and pons diameter values in groups divided by breed (Chihuahua, Maltese, Mixed, Pomeranian, Poodle, Shih Tzu, Yorkshire Terrier).

Breed	MH	MW	PH	PW
Chihuahua (*n* = 12)	7.25 ± 0.29	17.61 ± 1.07	9.97 ± 0.68	16.71 ± 0.73
Maltese (*n* = 59)	7.00 ± 0.58	17.52 ± 1.24	9.84 ± 0.68	17.02 ± 1.32
Mixed (*n* = 18)	7.25 ± 0.46	17.15 ± 1.00	9.67 ± 0.70	17.86 ± 1.47
Pomeranian (*n* = 35)	7.34 ± 0.37	16.93 ± 1.15	9.35 ± 0.57	16.90 ± 1.00
Poodle (*n* = 33)	7.42 ± 0.55	17.70 ± 1.09	9.85 ± 0.62	17.81 ± 1.15
Shih Tzu (*n* = 20)	6.71 ± 0.70	17.60 ± 1.54	9.69 ± 0.59	17.07 ± 0.96
Yorkshire Terrier (*n* = 16)	7.41 ± 0.35	17.51 ± 1.14	9.83 ± 0.48	17.38 ± 1.13

MH, midbrain height; MW, midbrain width; PH, pons height; PW, pons width.

**Table 4:** Mean and standard deviations of medulla oblongata diameter values in groups divided by breed (Chihuahua, Maltese, Mixed, Pomeranian, Poodle, Shih Tzu, Yorkshire Terrier).

Breed	MOHV	MOHC	RMOW	CMOW
Chihuahua (*n* = 9)	5.95 ± 0.72	5.83 ± 0.38	18.61 ± 0.67	9.96 ± 1.25
Maltese (*n* = 35)	5.87 ± 0.52	5.60 ± 0.38	18.39 ± 0.79	9.68 ± 0.84
Mixed (*n* = 11)	6.49 ± 0.30	5.92 ± 0.40	19.43 ± 1.47	10.16 ± 0.74
Pomeranian (*n* = 14)	6.07 ± 0.49	5.58 ± 0.38	18.66 ± 1.25	9.87 ± 1.20
Poodle (*n* = 22)	6.39 ± 0.35	6.07 ± 0.41	19.52 ± 1.45	10.83 ± 1.25
Shih Tzu (*n* = 19)	6.02 ± 0.51	5.79 ± 0.31	19.44 ± 1.47	10.27 ± 1.04
Yorkshire Terrier (*n* = 9)	5.69 ± 0.47	5.76 ± 0.34	18.67 ± 0.91	10.23 ± 0.80

MOHV, medulla oblongata height at the fourth ventricle level; MOHC, medulla oblongata height at the CM junction; RMOW, rostral medulla oblongata width; CMOW, caudal medulla oblongata width.

### 3.4. Intra-observer and inter-observer reliability

Observer A measured all values of the brainstem diameter twice. There was excellent reliability for the two measurements in all the brainstem diameters. The ICC for each brainstem diameter was: 0.992 (95% CI: 0.990–0.994) in MH; 0.981 (95% CI: 0.975–0.986) in MW; 0.992 (95% CI: 0.990–0.994) in PH; 0.983 (95% CI: 0.977–0.987) in PW; 0.997 (95% CI: 0.995–0.998) in MOHV; 0.992 (95% CI: 0.995–0.998) in MOHC; 0.984 (95% CI: 0.977–0.989) in RMOW; and 0.991 (95% CI: 0.987–0.994) in CMOW. The *p*-value for all values was <0.001.

All values were measured by two clinicians, observers A and B. There was excellent reliability for the two measurements in all the brainstem diameters. The ICC for each brainstem diameter was 0.945 (95% CI: 0.922–0.960) in MH; 0.942 (95% CI: 0.923–956) in MW; 0.959 (95% CI: 0.945–969) in PH; 0.968 (95% CI: 0.958–0.976) in PW; 0.886 (95% CI: 0.833–0.922) in MOHV; 0.907 (95% CI: 0.863–0.936) in MOHC; 0.946 (95% CI: 0.922–0.962) in RMOW; and 0.910 (95% CI: 0.836–0.946) in CMOW. The *p*-value for all values was <0.001.

## 4. Discussion

In this present study, we aimed to perform linear measurements of brainstem diameters, including the height and width of the midbrain, pons, and medulla oblongata. MR images of 193 dogs were evaluated and reference ranges of brainstem size in small-breed dogs were obtained. We present reference ranges of width and height for the midbrain, pons, and medulla oblongata for small-breed dogs.

This study found there was no significant difference between sexes in terms of brainstem diameters and this finding is in agreement with previous human studies (5, 10–14, 17, 18). In some human studies, there was only a difference in brainstem size between sexes in the age group of 50 or older (15, 19); another study demonstrated differences in midbrain or pons size; however, they did not categorize the groups by age (9, 20). In humans, differences in brainstem size between sexes may be attributed to the correlation with the larger total intracranial volume in males (15, 17). Significant brainstem shrinkage may be owing to intrinsic or extrinsic factors such as hormones, hypertension, or interactions with environmental factors, however, it has not been clarified thoroughly (15, 19). In the present study, mean values of MH, PH, and MOHV were slightly higher in females, whereas mean values of MW, PW, MOHC, RMOW, and CMOW were slightly higher in males, but this difference was not statistically significant. There was a significant difference in mean BW between males and females (larger in males). It was considered that there might be differences in the structure of the calvarium between the various breeds included in the sample population of this study (21, 22).

Results of the present study showed a positive correlation with BW; this is similar to the findings of previous studies of brain area (including the brainstem) in dogs (22, 23). The relationship between BCS and brainstem diameter was also considered in this study. BCS alone had no significant correlation with brainstem diameter. However, BW/BCS showed a positive correlation with brainstem diameter. The BCS may correlate negatively with brainstem diameter as obesity has a negative correlation with an index of physique (24). It is assumed that the reason for the lack of correlation between brainstem diameter and the BCS is the BCS of the dogs included for the measurement was mostly between four and six and the proportion of the dogs with extreme BCS was relatively low. As the BW/BCS index positively correlated with brainstem diameter, it could be more accurate to use the BW/BCS index in dogs with extreme BCS to evaluate normal ranges of brainstem size; however, using BW alone could be also accurate in dogs with an ideal BCS.

Only midbrain diameter (MH, MW) was negatively correlated with age, and no other values (PH, PW, MOHV, MOHC, RMOW, and CMOW) were significantly correlated with age. Several human imaging studies have reported a variation in the changes in different brain regions with age and volume loss with age depending on the brain area (5, 9–20, 25–28). Particularly in the human brainstem, a significant age-related decrease in midbrain area was confirmed (5, 9, 11–14, 16, 17, 19, 20, 29). For the pons and medulla, no significant correlation with age was identified (9–11, 13, 17–19, 27–29). Additionally, a minimal decline in the pons and medulla oblongata with aging was found in a few studies but the significance was not clear (5, 12). These aforementioned findings are in accordance with our results.

Physiological or pathological changes of the brain in aging dogs resemble the changes found in humans (21, 22, 30, 31). In dogs, cerebral atrophy, including findings such as widening of the cerebral sulci, ventriculomegaly, a decrease in frontal lobe volume, a decrease in interthalamic adhesion, and a decrease in the hippocampus, can be found in normal aging dogs (22, 23, 29–31). These changes may have multifactorial etiopathogenesis associated with β amyloid deposition, oxidative damage, decreased glycosphingolipids, neuronal shrinkage, neurotransmitter deficits, or decreased neurogenesis (21, 23, 30, 32). Although age changes of the brainstem have not been broadly studied in dogs, it has been demonstrated that hypointensity of the substantia nigra in the T2-weighted image is associated with iron accumulation resulting from the metabolism of neurotransmitters (31, 33–35). In the human brainstem, prominent age-related decline of midbrain size has been reported (5, 9, 11–14, 16, 17, 19, 20, 29), consistent with the present study. The midbrain has fibers and numerous nuclear masses in the tegmentum and a decrease in midbrain size has been attributed to neuronal death or degeneration (6, 15, 17, 29). Shrinkage of the substantia nigra with a decrease in the number of neurons has also been reported (12, 15, 17, 36). These aging changes in humans can be suggested as the reason for the result in this study, i.e., that the midbrain diameter shrinks with aging in dogs. Besides changing with age, brainstem size can decrease or increase depending on pathological changes. The size or diameter of the brainstem decreases through atrophy associated with neurodegenerative disorders, and these structural measurements play an important role in diagnosis in humans (6, 9, 37). Furthermore, some diffusely infiltrating tumors, such as gliomas, may be detected through an increase in size, without observable differences in signal intensities due to changes in relaxation times (16).

In the present study, there were significant differences in brainstem diameters between breeds. All values, except MW, were significantly different between breeds. There were a number of breeds that presented significant differences for each measurement, as shown in Figure 4. The shape or volume of the skull and brain structure vary depending on the breed or size of the dog (21, 22, 32, 38–40). Particularly, the Maltese differed from other breeds in most measurements, and most of the mean values, except PH, were significantly smaller than other breeds. This would have contributed to BW distribution, as the mean BW of the Maltese was relatively low compared with that of other breeds.

ICC with 95% CI was used in intra-observer and inter-observer reliability analyses. Interclass correlation was interpreted by criteria according to Fleiss (41): values <0.40 were considered poor agreement, values between 0.40 and 0.75 were considered fair to good agreement, and values >0.75 were considered excellent agreement (41). Intra-and inter-observer reliability analysis showed excellent agreement in all of the measurements in this study, indicating that there was no significant error in the measurement method of brainstem diameter.

Our study has some considerable limitations. First, owing to the limitations of retrospective study, the dogs used for measurement were not completely clinically healthy. However, we used brain MR images of patients with minimal brain lesions that were not clinically important and no brainstem lesions. Second, as this was a multicenter study, the location of the slice for the transverse plane was relatively diverse depending on the radiologist. Additionally, a particular anatomical structure was not used for the numerous measurement points. Therefore, the measurement points may not be perfectly accurate. Third, there was also a difference of 2.5 or 3 mm in slice thickness between the two local animal hospitals. The definitive MRI slice thickness in small animals has not yet been determined (30, 42). However, as a thinner slice allows better contour and less volume averaging than a thicker slice (30, 42, 43), measurements may not be precise as the availability of high-field MRI increased in veterinary medicine and high-quality neurological imaging is possible at 3.0 Tesla MRI (44, 45), using higher-field MRI could improve the accuracy of the measuring points. Fourth, the sample size was small for the comparison between breeds. Evaluation with a larger sample size may be required for each breed.

In conclusion, we assessed linear measurements of the brainstem diameter using MRI in small-breed dogs. Midbrain diameter and age were significantly negatively correlated. There was no significant difference between sexes in terms of brainstem diameter. In some breeds, there was a significant mean difference in brainstem diameters, except MW. Knowledge of brainstem diameters with normal variations related to aging and breeds can be valuable in determining pathological variations, such as neurodegenerative diseases and tumors.

## Data availability statement

The original contributions presented in the study are included in the article/supplementary material, further inquiries can be directed to the corresponding author.

## Ethics statement

The animal study was reviewed and approved by IACUC NON 2022-086. Written informed consent was obtained from the owners for the participation of their animals in this study.

## Author contributions

JK and HY: conception and design, drafting the article, and revising the article for intellectual content. JK, DK, S-SK, KL, and HY: data acquisition. JK: analysis and interpretation of data. JK, KL, and HY: final approval of the completed article. All authors contributed to the article and approved the submitted version.

## Funding

This research was supported by National University Development Project a Jeonbuk National University in 2022.

## Abbreviations

BW: body weight
BCS: body condition score
MR: magnetic resonance
MRI: magnetic resonance imaging
MH: midbrain height
MW: midbrain width
PH: pons height
PW: pons width
MOHV: medulla oblongata height at the fourth ventricle level
MOHC: medulla oblongata height at the cervicomedullary junction level
RMOW: rostral medulla oblongata width
CMOW: caudal medulla oblongata width
CM: cervicomedullary
ICC: intraclass correlation coefficient
CI: confidence interval
